# Anatomical and histological descriptions of the alimentary system, salivary gland, and Malpighian tubules of *Legnotus limbosus*, the bordered shieldbug (Geoffroy, 1785) (Heteroptera: Cydnidae)-light and electron microscopic studies

**DOI:** 10.1007/s00709-025-02077-7

**Published:** 2025-05-21

**Authors:** Nurcan Özyurt Koçakoğlu, Hicret Arslan, Selami Candan

**Affiliations:** Science Faculty, Department of Biology, Gazi University, Ankara, Turkey

**Keywords:** Digestive canal, Malpighian tubules, Histoanatomy, Light microscope, SEM

## Abstract

We reported the histological and anatomical analyses of *Legnotus limbosus*'s alimentary and excretory system using stereomicroscope, light and electron microscopy. As a result of the obtained data, the digestive tract of *L. limbosus* has three main parts: fore, mid, and hindgut. The salivary gland and gastric caeca are structures that assist digestion. The salivary gland is a pair consisting of the principal and accessory salivary glands. The foregut has the pharynx and esophagus. The pharynx structure is located immediately after the mouth and continues with the esophagus as a thin, long canal. The esophagus connects to the ventriculus 1 (V1). The midgut consists of three parts: V1, V2, and V3. The V1 and V2 have a single layer of cylindrical epithelium. However, the proximal and distal parts of V3 have cylindrical epithelium, while the lateral part exhibits a cuboidal form. The ileum and rectum make up the hindgut. The first has a cylindrical epithelium; the second has a squamous epithelium. The two pairs of Malpighian tubules, which are attached to the midgut-hindgut junction, are responsible for excretion and osmoregulation. Crystals with a deltoid shape are seen in the lumen of the Malpighian tubule and the rectum. This study is the first on the digestive and excretory system morphology of the Cydnidae family and will make significant contributions to studies on this subject in the Heteroptera, including this family.

## Introduction

Burrower bugs (Cydnidae) are among the most diverse families in the Pentatomoidea superfamily, second only to Pentatomidae. These insects are often called"burrower bugs"due to their habit of living in the soil and feeding on the sap from plant roots and basal parts of plants and seeds (Lis 1999). The family consists of 90 contemporary genera and about 700 species distributed across different zoogeographical regions around the globe (Lis 2012).

Burrower bugs are typical geo-herpetobionts (Mapкинa, 2017). Their adaptation to life in the soil is manifested in their digging leg structures and generally more complex heterotaxy (Киpичeнкo, 1951). Some members of the family can damage agricultural crops by feeding on the roots of vegetable plants, cereal crops, and fruit trees (Пyчкoв et al.^1961^; Пyчкoв, Oтpяд, 1972). In agriculture, the most harmful burrower bugs are often species from the Scaptocorini tribe in Central America (which damages banana plantations). However, some species have also been identified as agricultural pests in Western Europe, such as *Aethus nigritus* (Fabricius, 1794), *Sehirus luctuosus* Mulsant & Rey, 1866, *Tritomegas bicolor* (Linnaeus, 1758), and *T. sexmaculatus* (Rambur, 1839), which also occur in Belarus (Пyчкoв, Oтpяд, 1972). Therefore, the study of this taxonomic group, which is an integral part of natural communities and plays an essential role in human activities, is important not only from a scientific but also a practical standpoint.

Heteroptera species exhibit various feeding behaviors, including phytophagy, zoophagy, and hematophagy. This dietary diversity, coupled with the monophyletic nature of Heteroptera, makes these insects ideal subjects for studies of the digestive system (Santos et al. 2017).

The digestive process is separated into three parts in insects belonging to the order Heteroptera, with the mouth at the beginning and the anus at the finish. The midgut is where most digestion and nutrient absorption takes place; the hindgut is in charge of water absorption and homeostasis; and the foregut stores, filters, and partially digests food. The midgut is devoid of the cuticle that lines the foregut and hindgut (Candan et al. 2021; Dantas et al. 2021; Özyurt Koçakoğlu 2021; Özyurt Koçakoğlu and Candan 2021; Arslan and Candan 2025).

The salivary glands are in the thorax, on either side of the digestive tract. The structure of these glands can vary from comparatively simple to highly branching and complex. Two principal and accessory salivary glands comprise the salivary system in all Heteropteran species. Usually composed of anterior and posterior lobes, the principal glands are bilobed. Via its duct, the accessory salivary gland empties into the main salivary gland's hilus (Baptist 1941).

The oral cavity, pharynx, esophagus, proventriculus, gizzard (which grinds food), and intestine are commonly found in insects'foreguts. However, because their primary food source is plant sap, insects with piercing-sucking mouthparts, like those in the Heteroptera-Hemiptera group, do not possess a crop (Özyurt Koçakoğlu 2021). Often called the stomach or ventricle, the midgut is the most extended region of the digestive system. This is where most digestion occurs (Dadd 1970; Wigglesworth 1972; Gullan and Cranston 2005; Sarwade and Bhawane 2013). The midgut of Heteroptera is a lengthy tube demonstrating morphological and functional differentiation. It is the leading site for absorption and digestion and is also where different digestive enzymes are secreted (Billingsley and Lehane 1997; Fialho et al. 2009; Chapman 2013b, a). The gastric caeca is a pouch located at the front end of the midgut of the majority of insect groups. The leading site for the production of digestive enzymes and the absorption of digestion products is the gastric caeca (Chapman 1998). According to Maddrell and Gardiner (1980), Özyurt Koçakoğlu et al. (2020), Sarwade and Bhawane (2013), and Sinha (1958), the hindgut is lined with chitin and comes from the ectoderm. In insects, the hindgut is the last part of the digestive system and is usually separated into three parts: the rectum, colon, and ileum. However, the rectum and pylorus comprise the hindgut in Heteroptera (Gullan and Cranston 2005; Özyurt Koçakoğlu 2021; Arslan and Candan 2025).

Morphological analysis of the alimentary canal is crucial for understanding the fundamental structure of an organism. It provides valuable insights for comparative studies of digestive systems in species raised under varying environmental conditions, exposed to factors such as pesticides, or in closely related species. Consequently, understanding the digestive system structure of *Legnotus limbosus* is vital for developing effective control strategies against this insect. This study aims to better understand the digestive biology of Cydnidae species by examining the morphological, histological, and ultrastructural features of the digestive system of *L. limbosus*. This study lays the groundwork for further research into this species'biological control.

## Material and methods

### Insect and stereomicroscope

Twenty adult male and female *L. limbosus* specimens were collected from a field close to Kazan, Ankara, Turkey, in June 2024. Following collection, the materials were taken to the laboratory for analysis using light and scanning electron microscopes (SEM). The insects were dissected in a 0.1 M sodium phosphate buffer (pH 7.2) and sedated with ethyl acetate vapors before being observed and photographed using an Olympus SZX7 stereomicroscope (SM) (Özyurt Koçakoğlu et al. 2020).

### Light microscope

In order to conduct a histological analysis, ten specimens were first dissected and stored in 10% neutral formalin for a whole day. Following a tap water wash, 50%–99% ethanol solutions were used to gradually dehydrate the samples. After that, the tissues were cleansed in two rounds of xylene for fifteen minutes each. Following the transition from xylene to paraffin, the specimens were pierced with paraffin and then embedded in liquid paraffin at 65 °C. After that, the paraffin solidified at room temperature. Thin sections, 5–6 μm thick, were cut from the paraffin blocks using a Microm HM 310 microtome. These sections were stained with Hematoxylin–Eosin (H&E) and Mallory's trichrome, and images were captured using an Olympus BX51 light microscope (LM) (Özyurt Koçakoğlu et al. 2020).

### Scanning electron microscope

In order to prepare for SEM analysis, ten specimens were first fixed in 2.5% glutaraldehyde and then rinsed with sodium phosphate buffer (pH 7.2). After being cleaned, the samples were dehydrated using ethanol solutions ranging from 50 to 99%. The samples were immersed in hexamethyldisilazane (HMDS) and then left to air dry. After drying, the samples were photographed whole before being fractured at specified points. The specimens were connected to stubs with double-sided adhesive tape, and then a thin layer of gold was applied under vacuum using a Polaron SC502 sputter coater. The observations and images were taken using the JEOL JSM 6060 LV SEM in Gazi University's electron microscope laboratory (Özyurt Koçakoğlu et al. 2020).

## Result

**Fig. 1:**
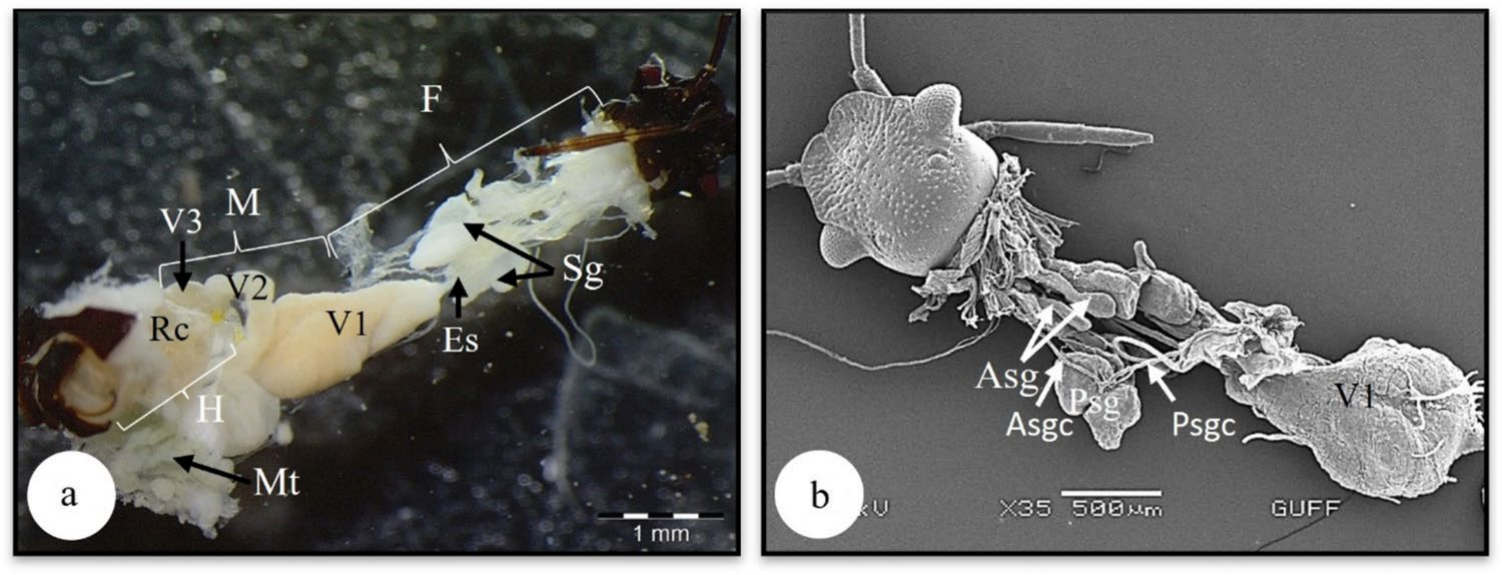
**a** General view of the digestive tract in *L. limbosus* (SM). **b** General view of salivary gland (SEM). Asg-accessory salivary gland, Asgc-accessory salivary gland canal, Es-esophagus, F-foregut, H-hindgut, M-midgut, Mt-Malpighian tubules, Psgc-principal salivary gland canal, Psg-principal salivary gland, Sg-salivary gland, V1-ventriculus 1, V2-ventriculus 2, V3-ventriculus 3

In *L. limbosus*, the alimentary canal starts with the mouth and ends with the anus and consists of 3 parts: foregut, midgut, and hindgut. The salivary gland and gastric caeca are structures that assist digestion (Fig. 1a). The organs under study in males and females did not differ from one another.

### The salivary gland and duct

**Fig. 2:**
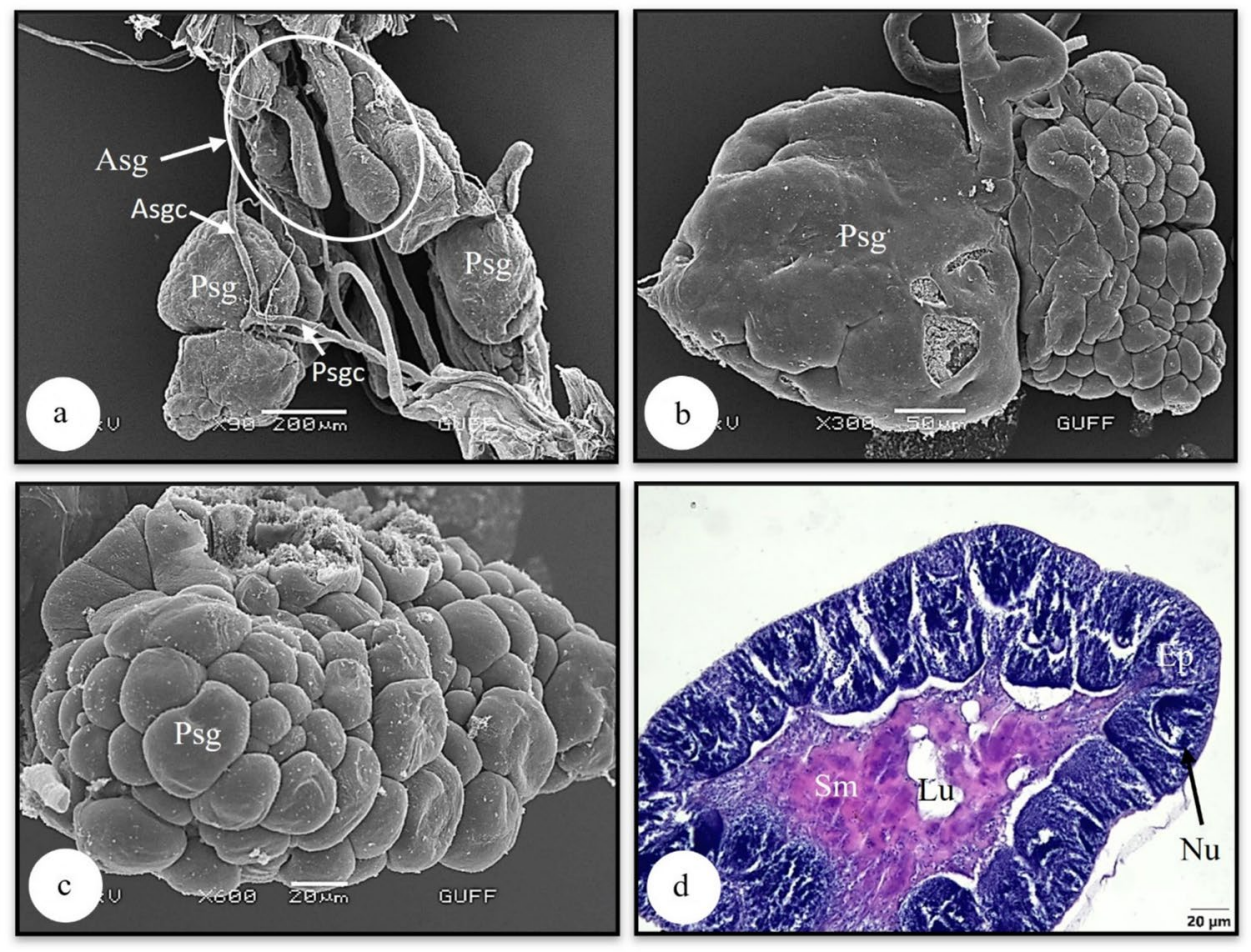
**a** SEM photograph of the parts forming the salivary gland. **b, c** Surface morphology of the principal salivary gland (SEM). **d** The histological section of the principal salivary gland (LM) (H&E). Asg-accessory salivary gland, Asgc-accessory salivary gland canal, Ep-Epithelium, Lu-lumen, Nu-nucleus, Psgc-principal salivary gland canal, Psg-principal salivary gland, Sm-secretion material, LM-light microscope, SEM-scanning electron microscope

**Fig. 3:**
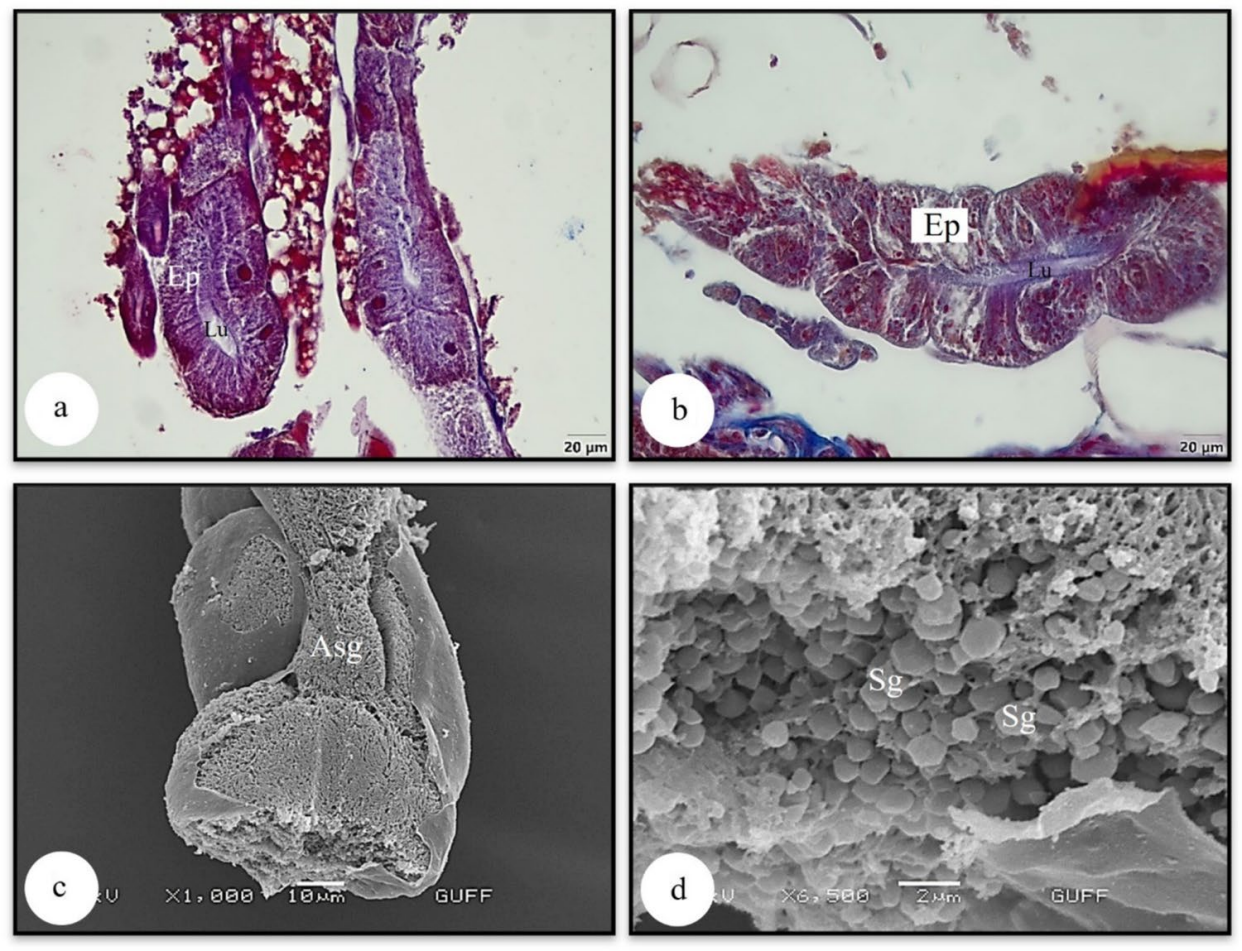
**a, b** The longitudinal section of accessory salivary gland (LM) (Mallory) **c, d** Secretory granules of the accessory salivary gland (SEM). Asg-accessory salivary gland, Ep-epithelium, Lu-lumen, Sg-secretory granule, LM-light microscope, SEM-scanning electron microscope

**Fig. 4:**
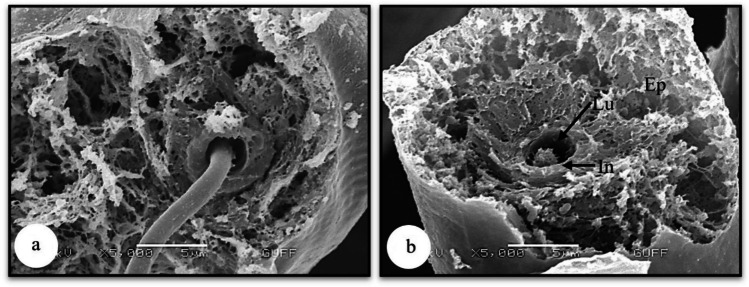
**a, b** Intima and epithelium surrounding the dried and transversely broken salivary duct (SEM). Ep-epithelium, Lu-lumen, In-intima, SEM-scanning electron microscope

The salivary gland consists a pair of the principal and accessory salivary glands. The salivary gland transfers its secretion to the oral cavity with the salivary duct, and the principal and accessory salivary glands each have a separate salivary duct (Figs. 1b, 2a). In the SEM examination of the principal salivary gland, it is seen that it has two lobes, one of which has a flat surface and the other has a budded appearance (Figs. 2b, c). In the histological section of the principal salivary gland, it is distinguished that nuclei of single-layered cuboidal epithelium with secretory granules and round nuclei surrounds it. The nucleus is dense with chromatin. The cytoplasm of epithelial cells shows a basophilic and granular structure. Its lumen is filled with eosinophilic secretory material (Fig. 2d). This gland, which transfers its secretion to the lumen, has an exocrine gland type. Another structure that forms the salivary gland, the accessory salivary gland, is in the form of a double tubular structure (Fig. 2a). Histologically, nuclei single-layered cuboidal epithelium with secretory granules and oval nuclei surround it. Figure 3b shows that the cytoplasm of the epithelial cells is filled with eosinophilic secretory granules. The lumen is relatively narrow (Figs. 3a, b). In SEM photographs of the accessory salivary gland, it is seen to be filled with secretory granules (Figs. 3c, d). In SEM photographs of the salivary duct into which the salivary gland opens, the thick intima layer surrounding the duct and the single-layered epithelium are distinguished (Figs. 4a, b).

### The foregut

**Fig. 5:**
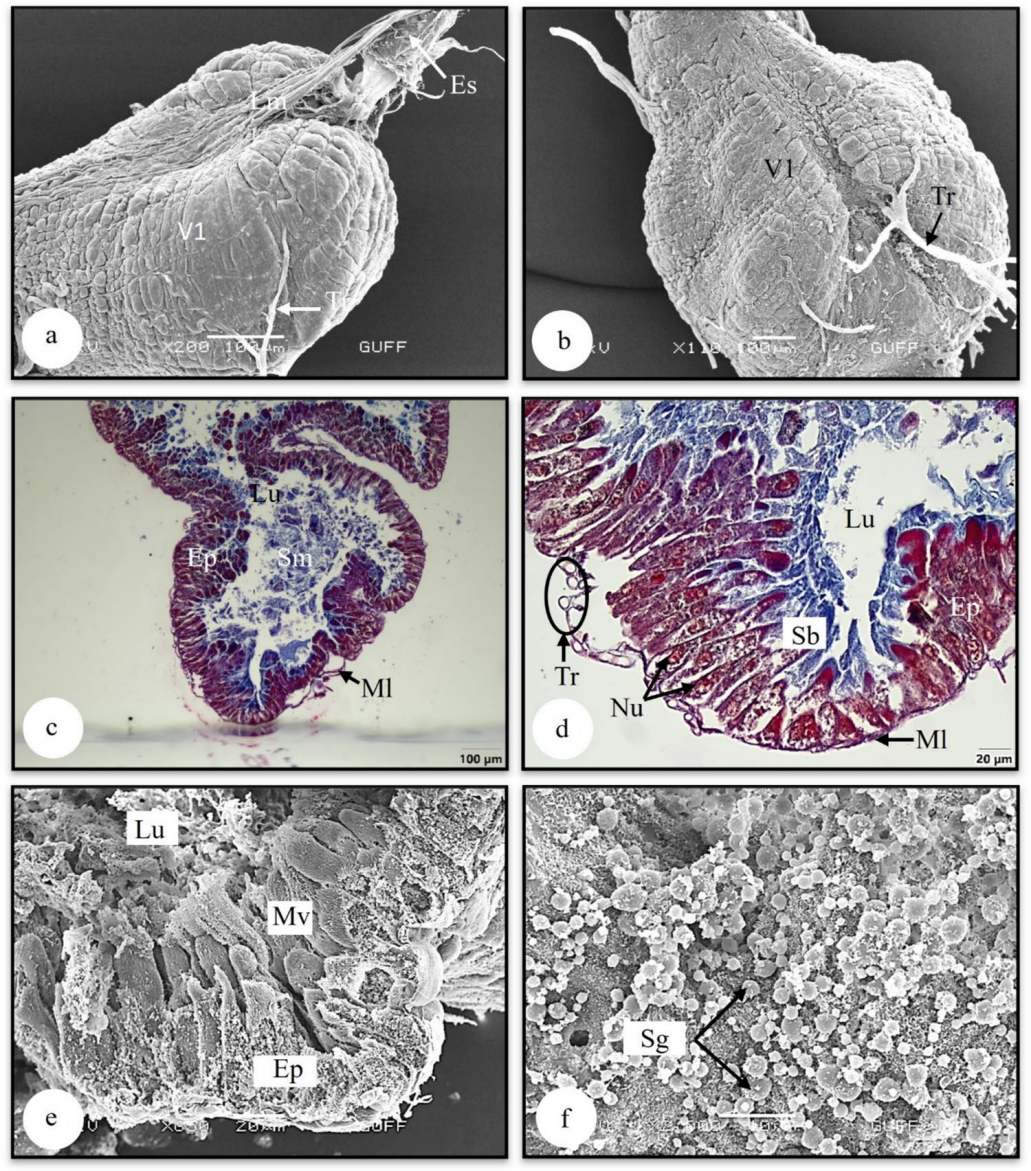
**a, b** Surface morphology of esophagus and ventriculus (SEM). **c, d** The longitudinal section of ventriculus 1 (LM) (Mallory). **e** The microvilli extending from the apical epithelium surrounding the wall of ventriculus 1 (SEM). **d** The secretory granules in the lumen of the ventriculus (SEM). Ep-epithelium, Es-esophagus, Lm-longitudinal muscles, Lu-lumen, Ml-muscle layer, Sm-secretory material, Tr-trachea, V1-ventriculus, LM-light microscope, SEM-scanning electron microscope

The foregut, the first part of the digestive system, consists of the pharynx and esophagus structures starting from the mouth (Figs. 1a, b). The pharynx structure is located immediately after the mouth and continues with the esophagus as a thin, long canal. Longitudinal muscles are noticeable in the SEM photograph of the esophagus (Fig. 5a). These muscles contract and transport food to the ventriculus (V1). V1 have a pear-shaped swollen appearance (Fig. 5b). In V1 SEM photographs, longitudinal and transverse muscles are noticeable on the surface (Figs. 5a, b).

### The midgut

**Fig. 6:**
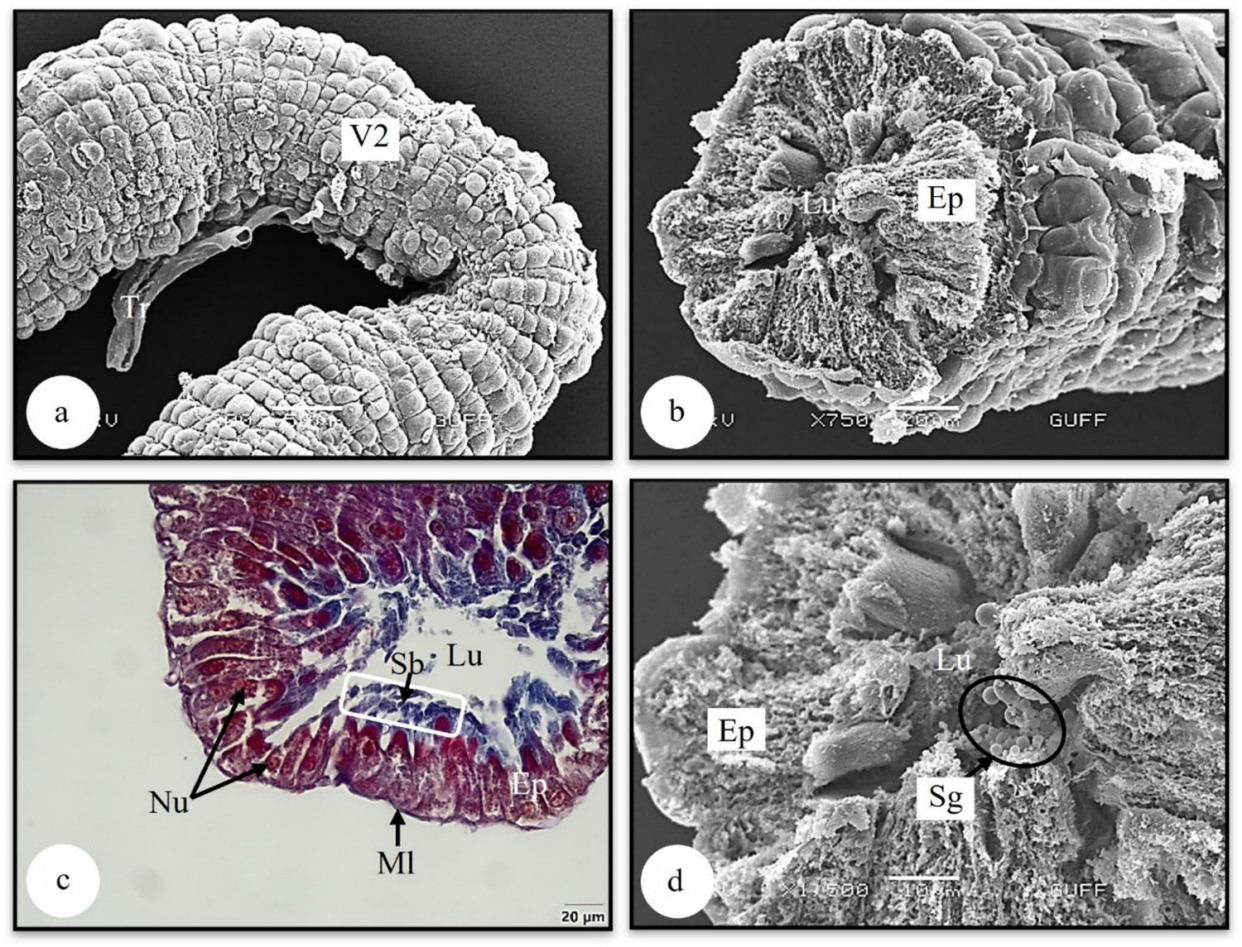
**a** Surface morphology of ventriculus 2 (SEM). **b** The epithelium surrounding ventriculus 2 (SEM). **c** The histological section of ventriculus 2 (LM) (Mallory). **d** The secretory granules in the lumen of ventriculus 2. Sb-striated border, Ep-epithelium, Lu-lumen, Ml-muscle layer, Nu-nucleus, Sg-secretory granules, Tr-trachea, V2-ventriculus 2, LM-light microscope, SEM-scanning electron microscope

**Fig. 7:**
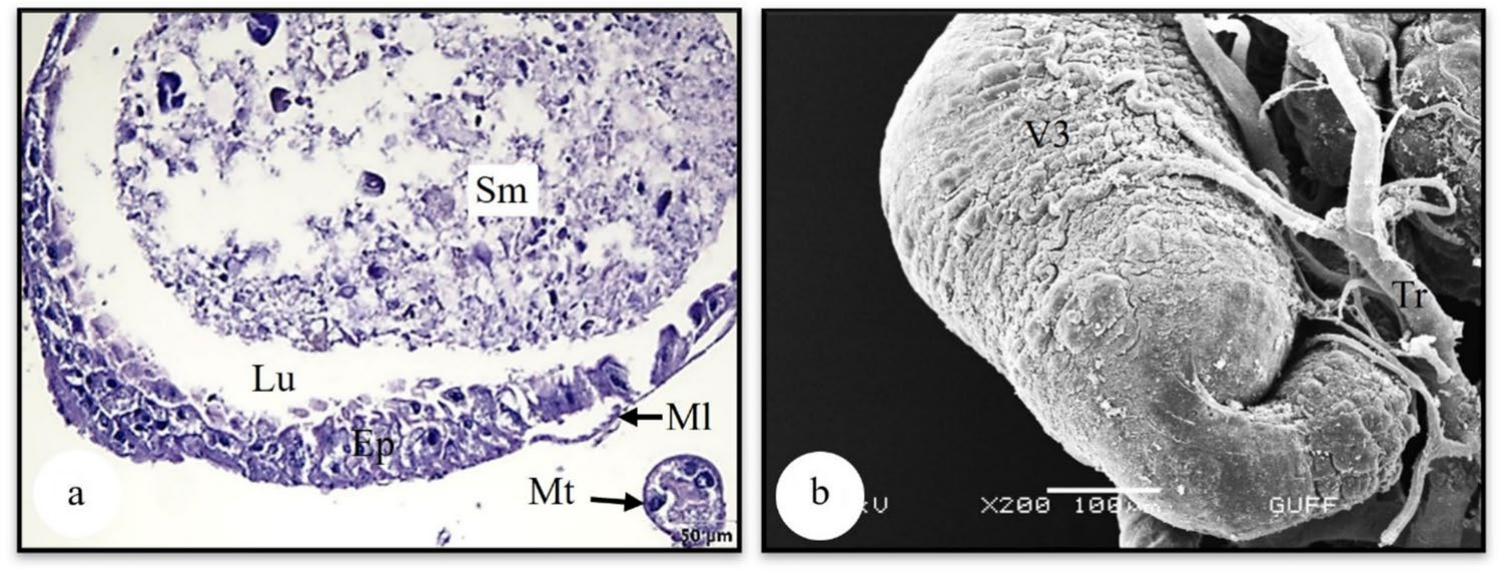
**a** The histological section of ventriculus 3 (LM) (H&E). **b** Surface morphology of ventriculus 3 (SEM). Ep-epithelium, Lu-lumen, Ml-muscle layer, Mt-Malpighian tubule, Sm-secretory material, Tr-trachea, V3-ventriculus 3, LM-light microscope, SEM-scanning electron microscope

The midgut, the second part of the digestive system, is the longest part of the digestive tract. The midgut structure consists of three parts: V1, V2, and V3 (Figs. 5b, 6a, 7b) In the light section of ventriculus 1 (V1), the single-layered cylindrical epithelial structure with oval nuclei has formed deep indentations towards the lumen. The epithelial cytoplasm shows eosinophilic characteristics (Fig. 5c). Basophilic secretory material is seen in the lumen (Figs. 5c, d). Figure 5d, striated border structures extending from the apical of the epithelial cells are noteworthy. A thin muscle layer was observed surrounding the epithelium from the outside (Fig. 5d). Microvilli structures responsible for absorption were observed at the apical of the epithelial cells (Fig. 5e). In Fig. 5f, secretory granules of different sizes are seen in the lumen of V1.

The second part of the midgut, V2, is ¼ the thickness of V1. The SEM photograph of the V2 surface structure was observed to resemble corn grains (Fig. 6a). In the SEM photographs and histological section of the dried and broken V2, it is seen that it is surrounded by a single layer of cylindrical epithelium that forms indentations towards the lumen and that the lumen is narrow (Figs. 6b, c). In Fig. 6c, it is distinguished that the epithelial cells have a granular structure and have nuclei with less dense chromatin. A striated border structure extends from the apical end of the epithelium. In addition, a thin muscle layer surrounds the epithelium (Fig. 6c). Secretory granules are seen in the lumen of V2 in (Fig. 6d).

It is distinguished that V3, which forms of the midgut's last part and is a swollen sac (Figs. 7a, b). Histologically, the lumen of V3 is filled with basophilic secretory material (Fig. 7a). It is surrounded by a single-layer epithelium and a thin muscle layer. While the epithelial cells have a cylindrical shape in the proximal and distal parts of V3, they are seen in a cubic form in the lateral part. Their nuclei are dense with chromatin (Fig. 7a). In the SEM photograph in Fig. 7b, the tracheal network connected to the surface of V3 is distinguished.

### The gastric caeca

**Fig. 8:**
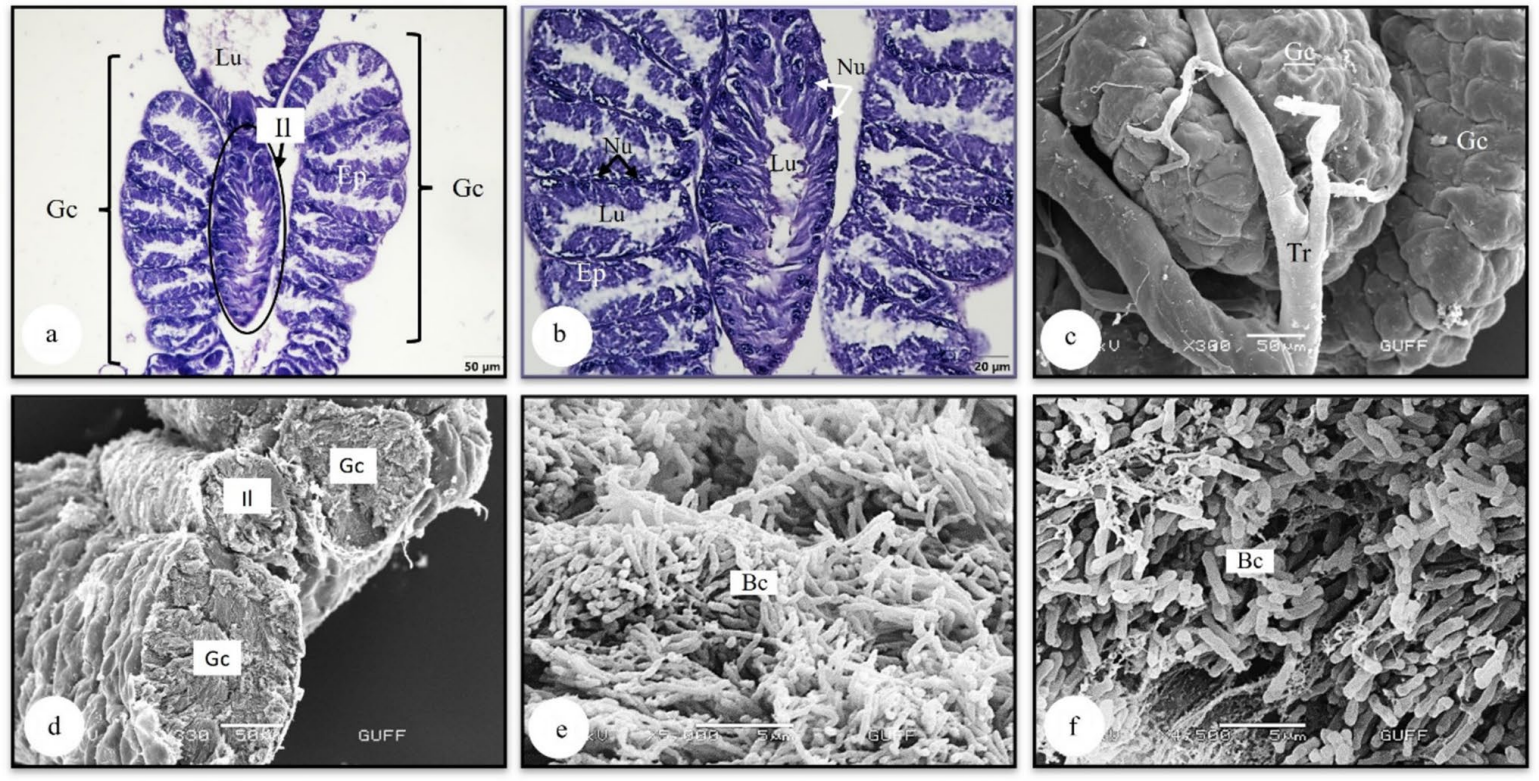
**a, b** The histological section of gastric caecum and ileum (LM) (H&E). **c** Surface morphology of gastric caecum (SEM). **d** Cross section of dried and broken gastric caecum and ileum (SEM). **e, f** Bacteria in the gastric caecum lumen (SEM). Bc-bacteria, Ep-epithelium, Gc-Gastric caeca, Il-ileum, Lu-lumen, Nu-Nucleus, Tr-trachea, LM-light microscope, SEM-scanning electron microscope

Gastric caeca are structures that help increase the absorption function of the midgut. They are tube-shaped structures that open into the ileum in the middle of the gastric caecum (Figs. 8a, b, d). In histological sections of the gastric caecum, it was observed that it consisted of oval structures resembling a series of coins, surrounded by a single-layered cylindrical epithelium and that the nucleus was pulled to the base of the cell due to the bacteria-filled interior (Figs. 8a, b). In the SEM photograph in Fig. 8c, the convoluted structure of the gastric caecum and the trachea connected to the surface is also distinguished. In the photographs of the dried and broken gastric caecum, it was observed that the content was filled entirely with bacillus bacteria (Figs. 8e, f).

### The hindgut

**Fig. 9:**
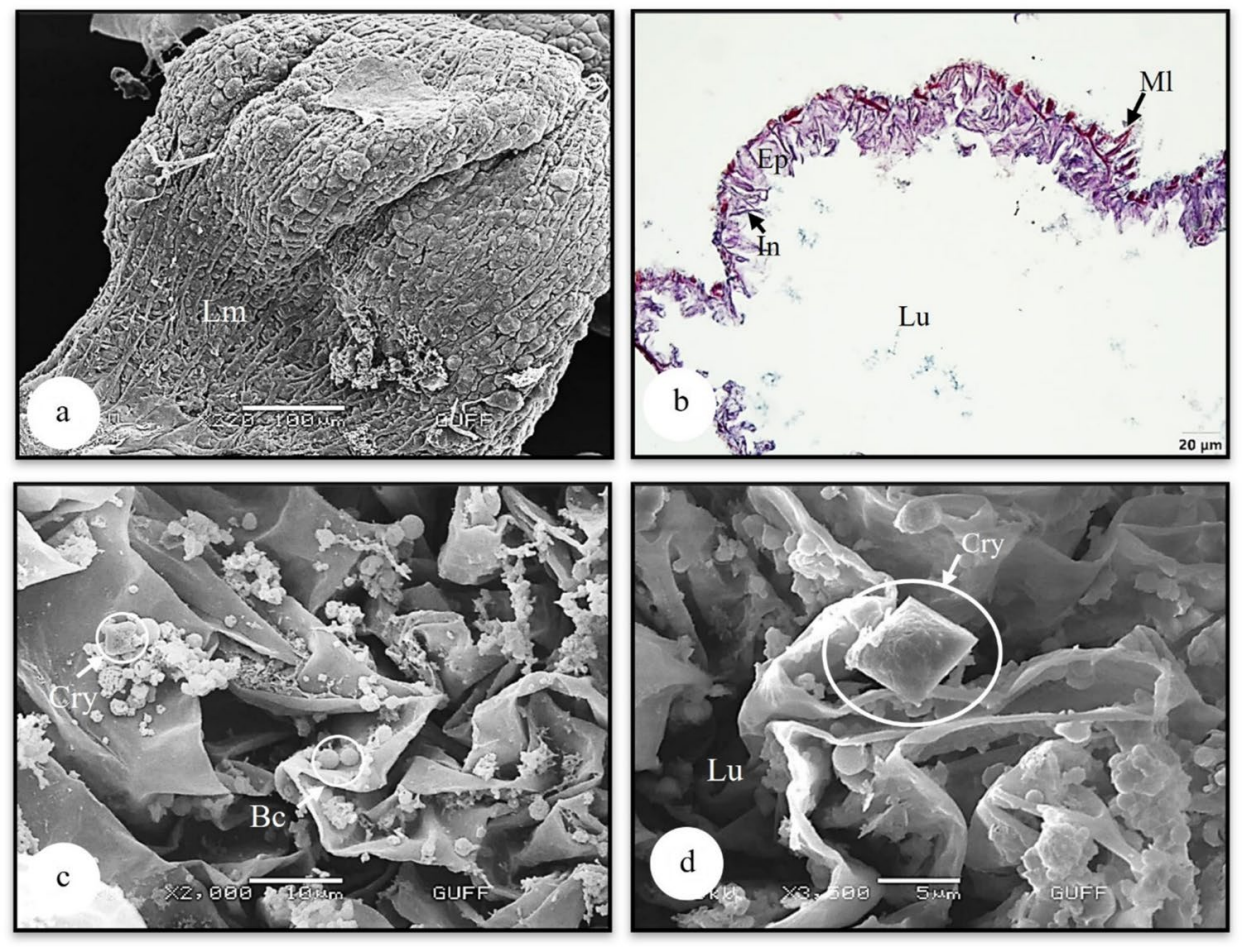
**a** Surface morphology of rectum (SEM). **b** The histological section of rectum (LM) (Mallory). **c, d** Bacteria and crystals in the gastric caecum lumen (SEM). Cry-crystal, Ep-epithelium, In-intima, Lm-longitudinal muscles, Lu-lumen, Ml-muscle layer, Bc-Bacteria, LM-light microscope, SEM-scanning electron microscope

The hindgut, the last part of digestion, consists of the ileum and the rectum. In the longitudinal section of the ileum, it is observed that it is surrounded by a single-layered cylindrical epithelium with oval and chromatin-dense nuclei (Figs. 8a, b). In the SEM photograph of the surface of the rectum, which is the second part of the hindgut, it is seen that it is a bulging, pouch-like structure surrounded by longitudinal muscles (Fig. 9a). In the histological section of the rectum, starting from the lumen, intima, single-layered flat epithelium and muscle layer are distinguished (Fig. 9b). In Fig. 9b, it is seen that the intima layer has sharp indentations. In the SEM photographs of the lumen of the dried and broken rectum, deltoid-shaped crystals and bacteria were observed (Figs. 9c, d).

### Malpighian tubules

**Fig. 10:**
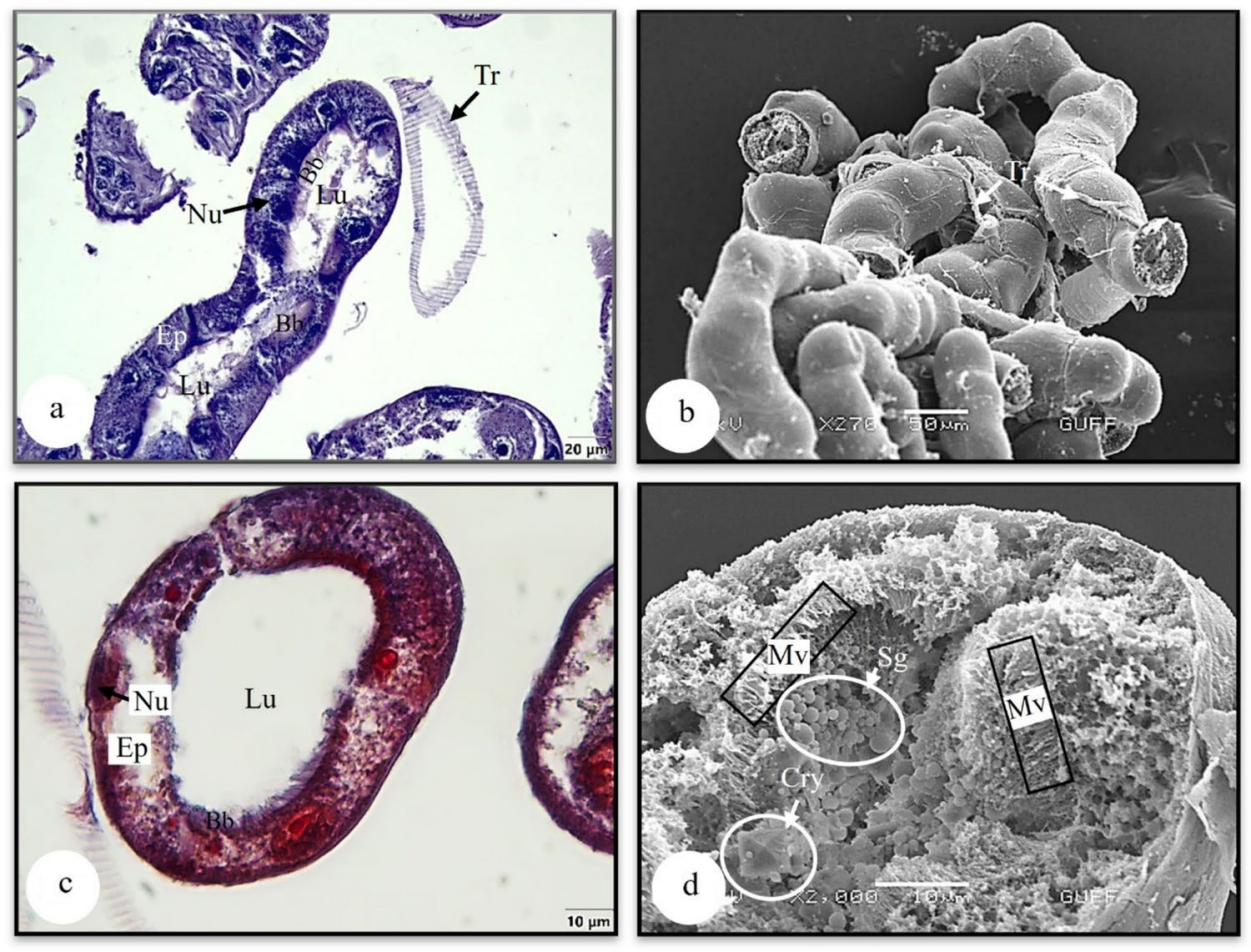
**a** The longitudinal section of Malpighian tubule (LM) (H&E). **b** The general view of Malpighian tubules (SEM). **c** The cross section of Malpighian tubule (LM) (Mallory). **d** Crystals and secretory granules in the lumen of Malpighian tubules (SEM). Bb-brush border, Cry-crystal, Ep-epithelium, Lu-lumen, Mv-microvilli, Nu-nucleus, Sg-secretory granules, Tr-trachea, LM-light microscope, SEM-scanning electron microscope

Malpighian tubules are two pairs and are structures responsible for excretion and osmoregulation connected to the junction of the midgut and hindgut (Fig. 1a). The distal end of these tubules is free in the hemosol. It forms a wavy form with a beaded end (Fig. 10b). Histologically, it was observed in the longitudinal and cross-sections of the Malpighian tubules that they are surrounded by granulated single-layered cuboidal epithelium with round and chromatin-dense nuclei (Figs. 10a, c). Figure 10 shows that short microvilli are noticeable at the apical part of the cell (Fig. 10d). Secretory granules of different sizes and deltoid-shaped crystals were found in the lumen of these tubules (Fig. 10d).

## Discussion

Nearly every ecological niche that supports insect life is home to Hemiptera, which are unable to sustain themselves solely on dry, dead organic materials (although many species consume dry seeds, while others consume moist plant or animal remnants or excreta). As a result, they do not use stored goods or dry wood, although they do contain only insects that are pelagic on the ocean surface. This successful adaptive radiation implies that the Hemiptera have evolved a very beneficial characteristic of their physiology or structure (Goodchild 1966). Highly specialized suctorial mouthparts, which are structurally comparable throughout the order, are a defining characteristic of insects in the Hemiptera. On the other hand, their alimentary canals exhibit a considerable deal of diversity and, in certain groups, a tremendous deal of complexity (Goodchild 1966). The foregut, midgut, and hindgut are the three components that make up an insect's digestive system. Additionally, the alimentary system is related to tissues like the caecum and salivary glands (Chapman 1998). The alimentary system of *L. limbosus* is also similar to the general insect digestive system and it consists of the foregut, midgut and hindgut. In addition, a pair of salivary glands, the gastric caecum and two pairs of Malpighian tubules are connected to the digestive tract.

### The salivary gland and duct

Saliva secretion from the salivary gland provides a solvent effect for food and other substances, and also moistens the mouth parts. The morphological variation in the salivary glands of hemipteran species may indicate their diverse feeding behaviors (such as zoophagous, phytophagous, zoophytophagous, and hematophagous). In all Heteroptera, the salivary system of typically comprised of a pair of principal salivary glands, accessory salivary glands, a principal salivary gland duct, and accessory salivary gland duct. The salivary system of *L. limbosus* has a pair of principal and accessory salivary glands and ducts, as in *Solubea pugnax* (Pentatomidae) (Hamner 1936), *Rhodnius prolixus* (Reduviidae) (Anhê et al. 2021), *Carpocoris mediterraneus* (Pentatomidae) (Özyurt Koçakoğlu and Candan 2022), *Aelia rostrata* (Pentatomidae) (Genç and Candan 2024) and *Eurydema spectabilis* (Pentatomidae) (Arslan and Candan 2025). The lobular structure and form of the salivary glands can differ between species, as observed in Hemipteran species. In several species, the principal salivary glands may be segmented into multiple lobes or resemble clusters of grapes (Elson 1937). For instance, the principal salivary gland of *L. limbosus* consists of two lobes, similarly, in *E. spectabilis* (Pentatomidae), it has been reported to have two lobes (Arslan & Candan 2025). In *Metacanthus elegans* (Berytidae), *Chilacis typhae* (Lygaeidae), and *Gastrodes ferrugineus* (Lygaeidae), the principal salivary gland is divided into three lobes: anterior, posterior, and lateral (Baptist 1941). However, in some species, the number of lobes in the principal salivary gland varies. For example, *Pyrrhocoris apterus* (Pyrrhocoridae) possesses a quadrilobed principal gland with anterior, posterior, median, and lateral lobes (Baptist 1941; Özyurt Koçakoğlu 2021). There are notable morphological variations among species with regard to salivary glands. This diversity may be due to differences in the feeding type of the species.

In *L. limbosus*, a single layer of cuboidal cells surrounds the principal salivary gland wall. Similar structure was found in *P. apterus* (PyrrhocoridaePyrrhocoridae) (Özyurt Koçakoğlu 2021), *C. mediterraneus* (Pentatomidae) (Özyurt Koçakoğlu and Candan 2022), *A. rostrata* (Pentatomidae) (Genç and Candan 2024) and *E. spectabilis* (Pentatomidae) (Arslan and Candan 2025).

The wall of the accessory salivary gland in *L. limbosus* is surrounded by a single layer of cuboidal epithelium, with numerous secretory granules present in the epithelium. A similar structure was observed in *P. apterus* (Pyrrhocoridae) (Özyurt Koçakoğlu 2021), *C. mediterraneus* (Pentatomidae) (Özyurt Koçakoğlu and Candan 2022), and *E. spectabilis* (Pentatomidae) (Arslan and Candan 2025).

## The foregut and midgut

In *L. limbosus*, as in *Oncopeltus fasciatus* Dallas (Heteroptera Lygaeidae), the foregut consists of the pharynx and esophagus. The esophagus is narrow, and tubular, opening into the enlarged first ventriculus (Al-Sandouk 1977).

In *L. limbosus*, the ventriculus 1 (V1) have a pear-shaped, swollen appearance. The ventriculus exhibits a structure with indentations on its surface, forming a single-layer cylindrical epithelial arrangement. Nuclei were observed embedded within the epithelium. Comparable structures have been observed in *Carpocoris pudicus* (Pentatomidae) (Metin 2014), *Lygaeus equestris* (Lygaeidae) (Demirkol 2016), *Coreus marginatus* (Coreidae) (Kara 2020), *P. apterus* (Pyrrhocoridae) (Özyurt Koçakoğlu 2021), *A. rostrata* (Pentatomidae) (Genç and Candan 2024) and *E. spectabilis* (Pentatomidae) (Arslan and Candan 2025).

The midgut is an important part of the insect digestive system. Along with other physiological functions, it is essential for metabolism, immunological response, electrolyte homeostasis, osmotic pressure, and circulation (Takeda 2012). Digestive enzyme synthesis and secretion, as well as nutrient absorption, are actively involved by midgut cells (Chapman 2013b, a). The number of parts comprising the midgut varies across species. In *L. limbosus*, the midgut is divided into three sections. Likewise, *Pyrops candelaria* (Fulgoridae) (Cheung and Marshall 1982), *Brontocoris tabidus* (Pentatomidae) (Azevedo et al. 2007), and *E. spectabilis* (Pentatomidae) (Arslan and Candan 2025).

The form of the midgut epithelial cells differs across species. In *L. limbosus*, midgut cells are arranged in a monolayer. The first and second ventriculus consist of a single layer of cylindrical epithelium. However, the proximal and distal parts of the third ventriculus have cylindrical epithelium, while the lateral part exhibits a cuboidal form. A single layer of cuboidal and cylindrical cells makes up the midgut epithelium of *Philaenus leucophthalmus* (Aphrophoridae) (Cecil 1930). In *E. spectabilis* (Pentatomidae), the first and second ventriculus comprise a single layer of cylindrical epithelium (Arslan and Candan 2025). Similarly, in *P. apterus* (Pyrrhocoridae), single-layered cylindrical and cuboidal epithelial structures have been noted. The first ventriculus contains cylindrical epithelium, while the third ventriculus comprises a cuboidal epithelial layer (Özyurt Koçakoğlu 2021).

## The gastric caeca

Glasgow (1914) noted the existence of well-developed caecal extensions at the posterior end of the digestive portion of the intestine while comparing the structure of the gastric caeca of Heteroptera species with that of other insects. These extensions had phylogenetic significance, he added. Bacteria were consistently shown to be present inside the gastric caeca. In Hemiptera, the gastric caeca are borne on a tubular region which follows the posterior bulb, the length of which varies from a small fraction to as much as three-quarters of the total midgut (Goodchild 1966). It has been stated that the gastric caecum increases digestive enzyme secretion and nutrient absorption (Chapman 2013b, a). In *L. limbosus*, the gastric caeca are a segmented structure composed of a single layer of cylindrical epithelium. Similar structures have been found in *E. spectabilis* (Pentatomidae) (Arslan and Candan 2025), *C. pudicus* (Pentatomidae) (Metin 2014), *L. equestris* (Lygaeidae) (Demirkol 2016), and *C. marginatus* (Coreidae) (Kara 2020). However, gastric caeca are absent in the midgut of *Eurygaster integriceps* (Scutelleridae) (Mehrabadi et al. 2012). It has been reported that the gastric caeca of *Nezara viridula* (Pentatomidae) (Hirose et al. 2006), *C. pudicus* (Pentatomidae) (Metin 2014), *L. equestris* (Lygaeidae) (Demirkol 2016), and *C. marginatus* (Coreidae) (Kara 2020) contain a significant number of digestive bacteria. We also found that *L. limbosus* was full of bacteria in its gastric caeca. However, no bacterial presence was found in the gastric caeca of *E. spectabilis* (Pentatomidae) (Arslan and Candan 2025).

## Malpighian tubules and hindgut

The Malpighian tubules primitively open into the extreme posterior end of the midgut in Hemiptera (Goodchild 1966). Malpighian tubules are excretory organs that function for the transport, metabolism and detoxification of dissolved organic substances. The number of Malpighian tubules differs significantly across species. *L. limbosus* has four Malpighian tubules, similar to *Triatoma infestans* (Reduviidae) and *E. spectabilis* (Pentatomidae) (Mello and Dolder 1977; Arslan and Candan 2025). However, *E. integriceps* (Scutelleridae) also have between four and six Malpighian tubules (Mehrabadi et al. 2012).

In histological studies, the epithelial structure of the Malpighian tubules in *L. limbosus* consists of a single layer of cuboidal epithelium, similar to that of *P. apterus* (Pyrrhocoridae), *C. mediterraneus* (Pentatomidae), *A. rostrata* (Pentatomidae) and *E. spectabilis* (Pentatomidae) (Özyurt Koçakoğlu 2021; Özyurt Koçakoğlu and Candan 2022; Genç and Candan 2024; Arslan and Candan 2025). A noticeable shift in epithelial type takes place at the level of the Malpighian tubule openings in many species (such as Fulgoroidea and Amphibicorisae), while in other groups (such as Coccoidea and Pentatomomorpha), the normal midgut epithelium extends past this point to the pyloric valve (Goodchild 1966).

We observed secretory granules and crystals in the Malpighian tubule lumen of *L. limbosus*, as in *C. mediterraneus* (Pentatomidae) and (Özyurt Koçakoğlu and Candan 2022). Secretory granules were observed in the epithelial cells surrounding the Malpighian tubule of *E. spectabilis* (Pentatomidae) (Arslan and Candan 2025).

In most insects, the hindgut makes some contribution to osmoregulation and plays an important role in homeostasis by regulating the reabsorption of water, salt, and other beneficial substances from the feces. In *L. limbosus*, the hindgut is examined in two parts: the ileum and the rectum, as in *P. apterus* (Pyrrhocoridae), *C. mediterraneus* (Pentatomidae) and *E. spectabilis* (Pentatomidae) (Özyurt Koçakoğlu 2021; Özyurt Koçakoğlu and Candan 2022; Arslan and Candan 2025).

Histologically, the shape of the cells surrounding the rectum differs across species, exhibiting cylindrical, cuboidal, and flat forms. In *L. limbosus*, the rectal wall is lined with a single layer of squamous epithelium. Likewise, in *C. mediterraneus* (Pentatomidae), the rectal wall is composed of a single layer of squamous epithelium (Özyurt Koçakoğlu and Candan 2022). On the other hand, in *P. apterus* (Pyrrhocoridae) and *E. spectabilis* (Pentatomidae), the cells around the rectum consist of cuboidal epithelial cells (Özyurt Koçakoğlu 2021; Arslan and Candan 2025).

In *L. limbosus*, abundant uric acid crystals were found in the rectum lumen. Similarly, they have been observed in *C. mediterraneus* (Pentatomidae) and *E. spectabilis* (Pentatomidae) (Özyurt Koçakoğlu and Candan 2022; Arslan and Candan 2025). Additionally, uric acid crystals were observed in *C. pudicus* (Pentatomidae), *R. nebulosa* (Pentatomidae), *L. equestris* (Lygaeidae), and *A. rostrata* (Pentatomidae) (Metin 2014; Bayramova 2015; Demirkol 2016; Genç, 2017). In *L. limbosus* rectum lumen, bacteria were found as like in *C. mediterraneus* (Pentatomidae) and *E. spectabilis* (Pentatomidae) (Özyurt Koçakoğlu and Candan 2022; Arslan and Candan 2025). The crystal seen in the lumen of the rectum of *L. limbosus* is deltoid-shaped as in *C. mediterraneus* (Pentatomidae), but the crystal in the lumen of the rectum of *E. spectabilis* has a round and radial form (Özyurt Koçakoğlu and Candan 2022; Arslan and Candan 2025).

In conclusion, in this research, we initially outlined the morphology and histology of the digestive tract of *L. limbosus*, highlighting its similarities and differences with other species. Through this, we aimed to contribute to systematic research. Moreover, this study is anticipated to enrich the understanding of the digestive system in Hemiptera.

## Data Availability

The data supporting this study's findings are available in this article's supplementary material.

## Declarations

### Additional information

This article was initially submitted by the authors in English and was first published here.
